# Effectiveness of the Smart Home Model for Integrated Malaria Prevention: A Case–Control Study in Oyam District, Uganda

**DOI:** 10.1155/jotm/6299085

**Published:** 2026-07-14

**Authors:** Anastasia Troia, Annalisa Rosso, Giovanni Cioni, Giovanna Letizia Calò, Lamberto Manzoli, Maria Elena Flacco

**Affiliations:** ^1^ Department of Environmental and Prevention Sciences, University of Ferrara, Ferrara, 44121, Italy, unife.it; ^2^ Department of Public Health and Infectious Diseases, Sapienza University of Rome, Roma, 00185, Italy, uniroma1.it; ^3^ Section of Hygiene and Preventive Medicine, University of Bologna, Bologna, 40126, Italy, unibo.it

**Keywords:** case–control study, integrated approach, integrated vector management, malaria prevention, multiple methods, primary prevention, Uganda, vector control

## Abstract

Malaria is the most prevalent vector‐borne disease worldwide, with Uganda ranking third for malaria burden in 2023. In resource‐limited settings, where access to diagnosis and treatment is often constrained and recent vaccination strategies may face challenges, traditional primary prevention remains essential and cost‐effective. This study evaluated the effectiveness of the smart home model—an integrated malaria prevention strategy promoted by the Uganda Ministry of Health—in reducing infections among children aged < 5 years. A case–control study was conducted in Oyam District, Northern Uganda, between October 2023 and February 2024. Data from 3093 households were analyzed. Malaria status was assessed using rapid diagnostic tests. Household adherence to nine vector‐control measures defined the smart home score, categorized as low (0–3), intermediate (4–6), or high (7–9). Higher scores were significantly associated with lower malaria prevalence. An inverse association emerged between household score and infection status: households with scores ≥ 7 were associated with a 80% lower presence of infection, as compared to households with scores ≤ 3 (AOR: 0.21; 95% CI: 0.16–0.27). The most protective measures included consistent use of bed nets, closing windows and doors by 6 p.m., eliminating water reservoirs, adopting proper farming practices, and compound cleanliness. These findings may provide further evidence supporting integrated malaria control strategies, alongside vaccines deployment, particularly in low‐resource settings.

## 1. Introduction

Malaria, caused by protozoan parasites transmitted to humans by infected female *Anopheles* mosquitoes, is the most common vector‐borne disease, with an estimated 263 million cases and 597,000 deaths worldwide in 2023 [1]. The disease is highly endemic in Africa, and Uganda shows the third highest malaria burden globally, with more than 12 million cases per year [1], and 9% of the children aged below five years (mostly living in rural areas) testing positive [2].

Although malaria is curable if diagnosed and treated promptly [1], low‐resource countries face a number of challenges, such as limited access to rapid diagnostic tests [3–5], emerging drug resistance [6–11], and prohibitive costs of treatments [12, 13]. As such, primary prevention may play a fundamental role in these areas [14]. Although two malaria vaccines have been recently made available in endemic regions [15, 16], challenges in vaccine supply, economic and logistical issues (e.g., cold chain maintenance), and the complexity of a multiple‐dose schedule limit their widespread use in these areas [17]. Consequently, vaccines should be considered complementary to strategies focused on vector control and health education, which remain critical because of their simplicity and limited costs [18–20]. In this regard, since 2021, the Uganda Ministry of Health has actively pursued the so‐called smart home model, i.e., homes that adhere to specific housing standards outlined in the Ministry’s antimalaria checklist, based on the combination of multiple vector control strategies [21].

So far, several studies evaluated the association between housing type and malaria burden [22–27] and the impact of using integrated malaria prevention methods [28]. However, the available evidence has mainly focused on the use of insecticide‐treated nets (ITNs) and indoor residual spraying (IRS) [29–49], and the available evidence assessing the integrated role of multiple vector control strategies is still limited [50–55].

The aim of the present study is to assess the associations of the smart home model to prevent malaria infections among children under 5 years old and to quantify (a) the combined effect of up to nine malaria prevention strategies and (b) the specific contribution of each strategy in the context of a rural, high‐endemic area.

## 2. Materials and Methods

The present case–control study was carried out between October 2023 and February 2024, corresponding to one of the two rainy seasons of the year—when malaria infection rates tend to rise [56]—in four subcounties of the Oyam District (Aber, Acaba, Iceme, and Myene), Northern Uganda. The sample included households with children aged below 5 years; cases were defined as the households with malaria‐positive children, while controls were those with malaria‐negative children. Malaria infection status was determined using malaria rapid diagnostic tests (RDTs) [57–59]; data on test positivity were collected between October and December 2023 and were extracted from each Village Health Teams’ (VHTs) data records.

The primary exposure of interest was the degree of household healthiness, as smart home level, carried out in February 2024 by specifically trained members of the VHTs, community‐based volunteer health workers.

As no standardized measurement tool for the smart home initiative has been developed at the ministerial level, we constructed a composite score to quantify household adherence to the recommended malaria prevention measures. The score components were selected based on the official Ministry of Health guidelines for “Mass Action Against Malaria (MAAM)—Malaria Smart Homes Model,” specifically based on the indicators for indoor and outdoor malaria control activities listed under section “G: Malaria Smart Family/Household/Individual” [21]. Only feasible and applicable indicators within the rural context were included. Where possible, self‐reported data were cross‐validated through direct observation of household conditions by VHTs to mitigate social desirability bias. As this represents the first attempt to operationalize and measure the smart home model in a field context, all items were assigned equal weight.

The smart home data were collected using a specifically designed 12‐item questionnaire administered to participating households.

The questionnaire collected information on the family composition of the selected households and on nine specific home healthiness criteria, each evaluating the following: (1) the use of long‐lasting insecticidal nets (LLINs) every night; (2) the presence of functional mosquito screen of ventilators, windows, and doors; (3) the practice of closing windows and doors by 6 p.m.; (4) the application of IRS; (5) the covering of water reservoirs, such as jerrycans, saucepans, and water pots; (6) the use of drying racks and the proper disposal of stagnant water; (7) the adoption of malaria‐smart farming practices such as avoiding the planting of maize, banana, and sorghum, and not tying animals near the house; (8) the presence of mosquito‐repellent plants around the home; (9) the maintenance of a well‐kept compound, including a tidy rubbish pit and regularly cut grass. Each of the nine above‐mentioned criteria was treated as a dichotomic (yes–no) variable, and, for each household, a total score was computed, ranging from a minimum of zero (none of the smart home criteria fulfilled) to a maximum of nine (all criteria fulfilled). Based upon the total score, each household’s degree of healthiness was categorized into low (0–3 points), intermediate (4–6 points), and high (7–9 points).

### 2.1. Data Analysis

First, differences in the degree of healthiness and in each sociodemographic collected variable between households with malaria‐positive and households with malaria‐negative children were evaluated using the chi‐squared test for categorical variables, while Kruskal–Wallis test was used for nonparametric continuous variables (the distribution of the recorded continuous variables was assessed through the Shapiro–Wilk test).

Multivariate logistic regression was then used to assess the potential, independent predictors of positive malaria status among children. Two separate models were built: in the first, the smart home score was treated as an ordinal variable, and three score categories (low, intermediate, and high healthiness score) were included in the model as dummy variables. In the second model, the specific role of each item contributing to the smart home total score was separately evaluated. In both models, the number of household members (≥ 5 vs. < 5 members) and the number of children under 5 years per family (> 1 vs. 1) remained stable, as household crowding may affect the consistent use of preventive measures. As an additional side analysis, we also assessed the potential association between the smart home total score and each of the collected demographic and clinical variables.

In all analyses, statistical significance was defined as a two‐sided *p* value e <0.05, and all analyses were conducted using Stata, Version 13.1 (Stata Corp., College Station, TX, 2014).

## 3. Results

The sample included 3093 households, most of them living in the Acaba subcounty (62.9%). Of these, 54.3% (*n* = 1678) had malaria‐positive children (Table 1).

**TABLE 1:** Characteristics of the sample, overall and by malaria status among children aged < 5 years.

Variables	Overall sample	Positive tests	Negative tests	*p* ^∗^
	(*N* = 3093)	(*N* = 1678)	(*N* = 1415)	
Subcounty, %				< 0.001
Aber	10.8	12.0	9.4	
Acaba	62.9	60.9	65.3	
Iceme	18.3	22.2	13.7	
Myene	8.0	4.95	11.6	
Household members, %				0.07
≥ 5 members	60.6	62.0	58.8	
Children per family, %				< 0.001
> 1 child	38.1	47.7	26.7	
*Smart home* criteria, %				
LLIN^a^	87.5	81.9	94.1	< 0.001
Windows/doors screening	46.3	41.7	51.8	< 0.001
Closing windows/doors	77.8	70.9	85.9	< 0.001
IRS^b^	17.6	16.8	18.5	0.2
Covering water reservoirs	82.9	76.9	90.1	< 0.001
Drying rack	53.7	49.7	58.5	< 0.001
Farming practices	74.2	66.7	83.0	< 0.001
Repellent plants	57.3	52.0	63.6	< 0.001
Grass cutting	75.8	68.7	84.2	< 0.001
Smart home score				
Median score (IQR)	6.0 (4.0–7.0)	6.0 (4.0–7.0)	6.0 (5.0–8.0)	< 0.001
Score ≥ 7, %	42.7	37.0	49.5	< 0.001

*Note:* IQR: interquartile range.

^∗^Chi‐squared test for categorical variables; Kruskal–Wallis test for nonparametric continuous variables.

^a^Long‐lasting insecticidal nets.

^b^Indoor residual spray.

At univariate analyses, compared to the families with malaria‐negative children (controls), those with malaria‐positive cases included more commonly two (or more) children per family, and, in general, showed significantly lower degrees of adherence to almost all smart home criteria (all *p* < 0.001). Specifically, the largest differences between cases and controls were observed in the routine adoption of farming practices (66.7% vs. 83.0% among malaria‐positive and malaria‐negative families, respectively), grass cutting (68.7% vs. 84.2%), and closing windows/doors (70.9% vs. 85.9%; Table 1). Overall, the proportion of households reaching a smart home score ≥ 7 (and classified as highly healthy) was significantly lower among those with malaria‐positive children (37.0% vs. 49.5% for positive and negative households, respectively, *p* < 0.001; Table 1).

In adjusted, multivariable analyses, smaller families with one child (as compared to larger ones), as well as higher degrees of adherence to smart home criteria (scores ≥ 4), were inversely associated with testing positive for malaria. Specifically, an inverse association emerged between smart home scores and malaria test positivity: households with scores ≥ 7 and between 4 and 6 were associated with an 80%‐ and 75%‐lower presence of positive malaria tests, respectively, as compared to households with scores ≤ 3 (adjusted odds ratio [OR]: 0.21; 95% confidence interval [CI]: 0.16–0.27 and OR: 0.25; 95% CI: 0.19–0.31; Table 2, Model 1). When the analyses were repeated including all specific smart home criteria in the final model (Table 2, Model 2), an inverse association was observed only for the use of LLIN (OR: 0.44; 95% CI: 0.33–0.58), the habit of closing windows/doors by 6 p.m. (OR; 0.70; 95% CI: 0.56–0.88), covering water reservoirs (OR: 0.67; 95% CI: 0.53–0.85), farming practices (OR: 0.64; 95% CI: 0.52–0.78), and grass cutting (OR: 0.64; 95% CI: 0.52–0.79).

**TABLE 2:** Logistic regression: potential independent predictors of detecting a positive malaria test among children aged < 5 years.

Variables	Positive malaria test	*p*
	Odds ratio (CI 95%)	
Model 1:		
Household members, ≥ 5 vs. < 5	0.99 (0.85–1.16)	0.9
Children per family, > 1 vs. 1	2.51 (2.14–2.95)	< 0.001
Smart home score categories,		
Lowest 0–3 (ref. cat)	1	—
Medium 4–6	0.25 (0.19–0.31)	< 0.001
Highest 7–9	0.21 (0.16–0.27)	< 0.001
Model 2:		
Household members, ≥ 5 vs. < 5	0.94 (0.80–1.10)	< 0.001
Children per family, > 1 vs. 1	2.50 (2.11–2.92)	
Smart home criteria,		
LLIN^a^	0.44 (0.33–0.58)	< 0.001
Mosquito‐screening windows/doors	0.94 (0.79–1.11)	0.4
Closing windows/doors	0.70 (0.56–0.88)	0.002
IRS^b^	1.23 (0.99–1.51)	0.05
Covered water reservoirs	0.67 (0.53–0.85)	0.001
Drying rack	1.01 (0.86–1.20)	0.9
Farming practices	0.64 (0.52–0.78)	< 0.001
Repellent plants	0.95 (0.80–1.13)	0.5
Grass cut	0.64 (0.52–0.79)	< 0.001

*Note:* CI: 95% confidence interval. Multivariate logistic regression based upon 3093 households. Two different models were fit: (1) the first model included the *smart home* score, treated as a dummy variable, with higher score categories resulting from a higher number of adopted criteria; (2) the second model separately assessed each item included in the *smart home* score. In both models, the number of household members (≥ 5 vs. < 5 members) and the number of children under 5 years per family (> 1 vs. 1) remained stable.

^a^Long‐lasting insecticidal nets.

^b^Indoor residual spray.

In the model assessing the potential, independent association between selected sociodemographic variables and the level of smart home healthiness, family size and the presence of children positive for malaria were independently associated with the degree of home healthiness (Table 3). Specifically, compared to smaller families, those with ≥ 5 members were significantly more likely to report a higher level of home healthiness. In contrast, compared to families with malaria‐negative children, those with malaria‐positive children were significantly more likely to report a lower level of home healthiness (*p* < 0.001). No significant variation in the smart home healthiness score was observed with an increasing number of children per family.

**TABLE 3:** Multivariate analysis: association between selected demographic and clinical variables and smart home score variation.

Variables	Smart home score	*p*
	Coeff. (CI 95%)	
Household members, ≥ 5 vs. < 5	0.33 (0.17; 0.50)	< 0.001
Number of children per family, > 1 vs. 1	−0.13 (−0.30; 0.03)	0.1
Presence of ≥ 1 child with positive vs. negative test	−1.03 (−1.19; −0.87)	< 0.001

*Note:* A higher score denotes a higher degree of adherence to malaria protection criteria. Coeff.: coefficient; CI: 95% confidence intervals. Linear regression based on 3093 households.

## 4. Discussion

The main findings of this observational study are the following: first, among families living in rural, highly endemic areas, the thorough adoption of household prevention practices was inversely associated with malaria test positivity in children under 5 years of age, with higher adherence scores being associated with a lower presence of positive malaria tests. Second, among the suggested preventive strategies, the systematic use of bed nets, the habit of keeping doors and windows closed and of removing water reservoirs, the adoption of proper agricultural methods around the house, and maintaining a well‐kept compound showed the strongest association with a negative test. The present findings confirm the existing literature and support the adoption of multiple malaria prevention methods, as compared to the use of single methods, to achieve an additive effect in reducing malaria infection and mosquito density [33, 52, 60, 61]. However, achieving high adherence to all recommended measures may not be equally feasible across different contexts, given variations in cultural norms, socioeconomic capacity, environmental conditions, and housing structures [62]. Identifying the most effective and context‐sensitive strategies would therefore be essential to tailor integrated malaria prevention interventions to specific territories and populations, thereby maximizing their effectiveness and sustainability [63].

To date, a number of studies explored the additive role of multiple methods of malaria prevention in low‐ and middle‐income countries [28]; only few of these, however, specifically assessed the integrated role of simultaneously adopted preventive measures, beyond the widely used ITNs and IRS [50–53]. Additionally, the quantitative assessment of an integrated infection control strategy on malaria outcomes among children is based, to the best of our knowledge, upon very limited data (a total of 329 caregiver/child pairs, presenting with malaria in tertiary health institution in Benin city, Nigeria), aimed at evaluating malaria‐associated mortality and severity [54]. The present study was conducted on a largest population across multiple rural villages in Uganda and is among the first large‐scale studies to adopt a strictly quantitative approach to assess the burden of malaria infection among children, within the context of an integrated malaria control strategy. In addition, in support of the local Ministry of Health, this study developed an initial monitoring framework to quantify household adherence to the smart home model, including a household‐level integrated prevention scoring system.

In this context, it is very likely that the current scenario will be severely affected by the introduction of malaria vaccines in Uganda, most likely from April 2025, following the WHO recommendations [64]. It has to be considered, however, that malaria vaccine delivery and uptake within sub‐Saharan African contexts may face several barriers and challenges [65], including limited access to education and information [66–69], vaccine hesitancy [70–73], shortage of skilled healthcare workers, particularly in rural and remote areas [74], limited accessibility due to poor infrastructure and inadequate cold chain systems [17, 75], and unfavorable weather and geographical conditions [76]. Furthermore, insufficient funding [77, 78] and weak political commitment [79] may undermine the successful implementation and scale‐up of malaria vaccination programs [65].

For all these reasons, although the introduction of vaccines will be a significant step forward in malaria control strategies, traditional preventive measures will remain essential. These measures are particularly cost‐effective and sustainable, as they can be easily adopted by the broader population [80]. In contrast, vaccines may represent a significant financial burden for local administrations in resource‐limited countries in Africa [65]. Moreover, improving housing and the surrounding environment may play a pivotal role in simultaneously protecting all family members, with potential positive impact on neighboring communities [51, 81].

Therefore, particularly in low‐resource countries, vaccination strategies should be combined with integrated primary preventive measures in order to maximize protection against malaria resurgence and ensure a more comprehensive and sustainable public health impact [63, 82].

This study has some limitations that must be considered in interpreting its results. First, the control group included families with asymptomatic children who were not tested for malaria, potentially underestimating malaria prevalence among asymptomatic carriers [81]. Second, malaria testing could not be always performed, due to limited RDTs availability and financial constraints among VHTs, thus potentially affecting the accuracy and completeness of malaria prevalence estimates. These issues highlight the need to address the role of socioeconomic factors in future studies and interventions [83]. Third, an equal weighting of the nine preventive criteria was adopted, rather than a weighted system for each single criterion, as the score aimed to capture the overall adoption of complementary malaria prevention practices, consistent with the integrated vector management approach promoted by the WHO [60]. Furthermore, the retrospective nature of the study creates the risk that it may be prone to the potential limitation of reverse causality; indeed, households with malaria‐positive children may have changed behaviors after infection. However, structural and environmental characteristics of the household are typically slow to change in these rural settings due to cultural, economic, and logistical constraints. Therefore, it is highly unlikely that a household classified as having very high adherence to smart home criteria at the time of the survey would have had extremely low adherence only a few months earlier, or conversely [84].

Then, the regression models were not adjusted for certain potential confounders (socioeconomic status, maternal education, and housing materials) to maintain a simple, feasible data collection across a large, relatively homogeneous rural population.

Despite these limitations, the present study has several strengths: this is, to our knowledge, the first study based on a very large area across multiple subcounties of Uganda, specifically analyzing the impact of the smart home model, thus ensuring a high level of reliability and reproducibility to other contexts. Second, the questionnaire was designed to be short and simple, thus increasing the ease of administration by VHTs, also in highly deprived contexts, and facilitating the continuous monitoring of housing conditions, as well as regional and international comparisons.

In conclusion, the present study confirms the positive association between the use of multiple, combined preventive strategies and a low malaria prevalence rate among children, with high level of protection achieved in the contexts where all or almost all criteria were adopted. Although additional studies are strongly needed to evaluate their effectiveness in different settings and assess their operational feasibility, the present findings may provide useful insights toward an integrated malaria prevention model that can inform health policy and optimize resource allocation, particularly in economically disadvantaged areas.

## Author Contributions

Conceptualization: Anastasia Troia, Annalisa Rosso, Giovanni Cioni, Giovanna Letizia Calò, and Lamberto Manzoli; methodology: Annalisa Rosso, Maria Elena Flacco, and Lamberto Manzoli; software: Giovanni Cioni, Maria Elena Flacco, and Lamberto Manzoli; validation: Annalisa Rosso and Maria Elena Flacco; formal analysis: Anastasia Troia, Giovanna Letizia Calò, Maria Elena Flacco, and Lamberto Manzoli; investigation: Anastasia Troia, Giovanni Cioni, and Giovanna Letizia Calò; resources: Maria Elena Flacco, Annalisa Rosso, and Lamberto Manzoli; data curation: Anastasia Troia, Giovanni Cioni, and Giovanna Letizia Calò; writing–original draft preparation: Anastasia Troia and Maria Elena Flacco; writing–review and editing: Anastasia Troia, Annalisa Rosso, Maria Elena Flacco, and Lamberto Manzoli; visualization: Annalisa Rosso, Giovanni Cioni, and Giovanna Letizia Calò; supervision: Annalisa Rosso, Maria Elena Flacco, and Lamberto Manzoli; project administration: Anastasia Troia, Maria Elena Flacco, and Lamberto Manzoli; funding acquisition: none.

## Funding

This research received no external funding.

Open‐access publishing was facilitated by Universita degli Studi di Ferrara, as part of the Wiley–CRUI‐CARE agreement.

## Ethics Statement

The study was conducted in accordance with the principles of the Declaration of Helsinki. It did not request a formal evaluation by the Ugandan Ethics Committee, as the malaria prevention activities and efficacy evaluation were explicitly requested by the Ugandan Ministry of Health. Data collection was carried out among voluntary respondents after obtaining informed consent. To ensure data protection, all records were anonymized at the source, and only aggregated household‐level data were used for analyses. The study did not involve any clinical intervention or direct experimentation on patients.

## Conflicts of Interest

The authors declare no conflicts of interest.

## Data Availability

The data that support the findings of this study are available from the corresponding author upon reasonable request.
